# Inequalities in the prevalence of stunting, anemia and exclusive breastfeeding among African children

**DOI:** 10.1186/s12887-022-03395-y

**Published:** 2022-06-09

**Authors:** Michael Ekholuenetale, Osaretin Christabel Okonji, Chimezie Igwegbe Nzoputam, Amadou Barrow

**Affiliations:** 1 Department of Epidemiology and Medical Statistics, Faculty of Public Health, College of Medicine, University of Ibadan, Ibadan, Nigeria; 2 School of Pharmacy, University of the Western Cape, Cape Town, South Africa; 3 Department of Public Health, Center of Excellence in Reproductive Health Innovation (CERHI), College of Medical Sciences, University of Benin, Benin City, Nigeria; 4 Department of Medical Biochemistry, School of Basic Medical Sciences, University of Benin, Benin, Nigeria; 5 Department of Public & Environmental Health, School of Medicine & Allied Health Sciences, University of The Gambia, Kanifing, The Gambia

**Keywords:** Undernutrition, Child health, Infant, Breastfeeding, Malnutrition, Child nutrition

## Abstract

**Background:**

Childhood stunting and anemia are on the increase in many resource-constrained settings, without a counter increase in proper feeding practices such as exclusive breastfeeding. The objective of this study was to explore the prevalence of stunting, anemia and exclusive breastfeeding across African countries.

**Methods:**

Demographic and Health Survey (DHS) data from 39 African countries was analyzed. Data from under 5 children were analyzed. Forest plot was used to determine inequalities in the prevalence of the outcome variables.

**Results:**

The prevalence of stunting was highest in Burundi (56%), Madagascar (50%) and Niger (44%). In addition, Burkina Faso (88%), Mali (82%), Cote d’Ivoire and Guinea (75% each) and Niger (73%) had the highest prevalence of anemia. Furthermore, Burundi (83%), Rwanda (81%) and Zambia (70%) had the highest exclusive breastfeeding. We found statistical significant difference in the prevalence of stunting, anemia and exclusive breastfeeding (*p* < 0.001). Higher prevalence of stunting and anemia were estimated among the male, rural residents, those having mothers with low education and from poor household wealth.

**Conclusion:**

Concerted efforts are required to improve childhood health, survival and proper feeding practice. Reduced stunting and anemia could be achieved through sustained socioeconomic improvement that is shared in equity and equality among the population. Interventions aimed at increasing food availability can also aid in the reduction of hunger, particularly in impoverished communities.

## Background

Stunting is a common health problem among under 5 children in many resource-poor settings globally. It is defined as a deficit in height relative to a child’s age [1]. Approximately 165 million (26%) under 5 children were stunted in 2011, accounting for a 35% decrease from an estimated 253 million under 5 children in 1990 [2]. In 2016, an estimated 155 million (23%) children worldwide were stunted, with Africa accounting for more than one-third [3]. The high incidence of stunted children remains a serious public health issue, whereby more than 90% of the stunted under 5 children worldwide live in Africa and Asia. Malnutrition is thought to be responsible for nearly one-third of all childhood deaths [4]. In spite of the fact that problems linked to malnutrition affect the entire community, under 5 children are especially vulnerable due to their physiological peculiarities. Children can be vulnerable because of their need for proper feeding to enhance maturity or development of their cells, organs, systems and biomolecules to carry out the chemical and physical functions. Malnutrition reduction is critical for achieving the Sustainable Development Goals (SDGs), particularly those aimed at ending poverty in all forms everywhere (SDG 1), ending hunger, achieving food security, improving nutrition, and promoting sustainable agriculture (SDG 2) and ensuring healthy lives and promoting wellbeing for all at all ages (SDG 3) [5, 6]. Due to the negative impacts of childhood malnutrition, governments have agreed to set worldwide goals to reduce chronic undernutrition (stunting) by 40% by 2025, as well as to reduce the prevalence of acute undernutrition (wasting) in under 5 children to less than 5% [7].

In resource-poor settings, suboptimal feeding affects an enormous number of individuals [8]. This improper feeding habit has several adverse effects on the populace including, poor state of health, stunted growth, low productivity, intellectual deficiency and unexpected death. Suboptimal feeding can cause adverse health effects on under 5 children, precisely from the time of conception up to their first 24 months or within their first 1,000 days of life. These poor health conditions can lead to intellectual and physical consequences [9]. Suboptimal feeding is estimated to contribute up to 34% of childhood mortality each year [10, 11]. Besides, it can impede social and economic growth within any community, particularly in poor and infrastructural deprived settings. From Food and Agriculture Organization (FAO) report for 2011 and 2013, as much as 842 million persons globally could not obtain their nutritional energy requirements within these three years, in comparison with about 870 million persons projected between 2010 and 2012. By implication, one out of every eight persons is at risk of chronic hunger, which is defined as a lack of sufficient food for a healthy living. The staggering number of people without adequate food, approximately 827 million live in resource-poor settings, where undernutrition is currently estimated to reach 14.3% [8].

Anemia is defined as the below-normal red blood cell count or hemoglobin level per unit volume in peripheral blood [12]. Anemia among under 5 children is a public health problem worldwide. There are many causes of anemia, of the iron deficiency (inadequate iron intake, poor iron absorption or excess iron losses), insufficient hematopoiesis (such as from vitamin B-12 deficiency), loss of blood (hemorrhagic anemia), premature red blood cell plasma membrane rapture (hemolytic anemia), deficient or abnormal synthesis of hemoglobin (such as thalassemia) or destruction of bone marrow [13]. In addition, hidden hunger, can also cause anemia when micronutrient intake, such as vitamins and minerals, is insufficient [14]. The commonest micronutrient deficiency that affects as much as 2 billion people globally is Iron deficiency [15]. It has the potential of causing severe adverse effects on the neurodevelopment of children. Iron deficiency affects over 39% of under 5 children [16]. When this staggering estimates are not mitigated, it can develop to iron deficiency anemia, which can result to poor cognitive development among children [17, 18]. Iron deficiency has been reported as the prominent cause of anemia according to global burden of disease estimates from 187 countries [19]. Moreover, iron deficiency was the leading cause of anemia among women and children of preschool age, as reported from a survey on the burden of anemia among vulnerable groups [20]. Iron is fundamental for biological functioning of the human body, from seemingly inconsequential activities such as respiration and immunity, to more complex cellular activities such as DNA synthesis and cell proliferation [21, 22].

The standard for infant and child feeding as recommended by World Health Organization (WHO), comprises giving newborn only breast milk for the first 6 months of life [23, 24]. Though exclusive breastfeeding (EBF), as recommended by WHO, is to be practiced for the first 6 months of a baby’s life, however, breast milk is low in iron content [25]. Conversely, evidence-based studies have shown that giving a child exclusive breastfeeding is linked to large health benefits in child’s life and also leads to improvement of childhood survival [26, 27]. About a decade ago, the World Health Assembly in Resolution 65.6, endorsed an all-inclusive implementation plan for mothers, newborn and young children nutrition. The resolution specified 6 global nutrition targets for 2025, which include increasing the rate of exclusive breastfeeding up to 50% in the first six months of a child’s life [28]. A crucial component of infant growth processes is the support for proper breastfeeding practices. The International Code of Marketing of Breast Milk Substitutes [29], the Global Strategy for Infant and Young Child Feeding (IYCF) [30] as well as The Code, baby friendly hospital initiative (BFHI) [31], are among the global programmes designed to improve infant and child feeding practices. In recent years, WHO released a set of indicators to assess child feeding and track breastfeeding progress. Since then, there has been a lot of focus on infant and child feeding structures, as well as what constitutes good breastfeeding habits [23]. The objective of this study was to explore the prevalence of stunting, anemia and exclusive breastfeeding across African countries.

## Methods

### Data source

We analyzed cross-sectional secondary data from Demographic and Health Surveys (DHS) in African countries from 2002–2020. DHS adopts multi-stage cluster stratified sampling approach in data collection. The stratification method divides the respondents into groups based on their geographical location, which is frequently bridged by their place of residence: urban versus rural. To divide the population into first-level strata and subdivide the first-level strata into second-level strata, and so on, a multi-level stratification approach is utilized. In the DHS, the two levels of stratification are based on geographical region and urban/rural. The countries examined in this study include: Angola, Benin, Burkina Faso, Burundi, Cameroon, Chad, Comoros, Congo, Congo Democratic Republic, Cote d’Ivoire, Egypt, Eritrea, Eswatini, Ethiopia, Gabon, Gambia, Ghana, Guinea, Kenya, Lesotho, Liberia, Madagascar, Malawi, Mali, Morocco, Mozambique, Namibia, Niger, Nigeria, Rwanda, Sao Tome and Principe, Senegal, Sierra Leone, South Africa, Tanzania, Togo, Uganda, Zambia, Zimbabwe. DHS data is publicly available and can be found at http://dhsprogram.com/data/available-datasets.cfm.

Since 1984, over 85 countries have conducted this surveys, which are repeated every five years. A significant advantage of DHS is that the sampling design and data collection approach are consistent across countries, making ing results from various countries comparable. Despite the fact that the DHS was created to supplement the fertility, demographic, and family planning data collected in the World Fertility Surveys and Contraceptive Prevalence Surveys, it has quickly become the most important source of population surveillance for the monitoring of population health indices, especially in resource-constrained settings. DHS collects data on vaccination, child and maternal mortality, fertility, intimate partner violence, female genital mutilation, nutrition, lifestyle, infectious and non-infectious diseases, family planning, water and sanitation, and other health-related topics. DHS excels in gathering high-quality data by providing proper interviewer training, countrywide coverage, a consistent data collection instrument, and clear operational definitions of topics to help policymakers and decision-makers comprehend them. Data from the DHS can be used to create epidemiological studies that estimate prevalence, trends, and inequities. DHS's specifics have previously been revealed [32].

### Selection and measurement of variables

#### Outcome

Stunting: Children are termed stunted if their height-for-age Z-score (HAZ) is less than -2 standard deviations (-2 SD) from the WHO reference population median. Stunting refers to linear growth retardation and cumulative growth deficits in children, which is an effect of malnutrition [1].

Anemia is diagnosed by pricking the children's fingers or heels and measuring their hemoglobin levels. Anemia was dichotomized: anemic versus not anemic (< 11.0 g/dl versus 11.0 g/dl) respectively [20].

Exclusive breastfeeding refers to a measure used for infants under 6 months who were only fed with breastmilk [23]. This indicator was calculated using the meals of infants under 6 months in the 24 h prior to the survey.

### Independent variables

Child sex: male versus female; residential status: urban versus rural; mother’s education: no formal education/primary versus secondary/higher; birth interval: first birth, less than 24 months, 24–27 months, 48 + months; wealth quintiles: lowest, second, middle, fourth, highest. The wealth index was retained from the DHS as it is directly available in the dataset [33]. DHS household wealth index was calculated by constructing a linear index from asset ownership indicators using principal components analysis to derive weights. In the original survey, the wealth index was constructed by assigning household scores, then ranking each person in the household population by their score. Thereafter, the distribution was divided into five equal categories and each had 20% of the population with economic proxies, such as housing quality, household amenities, consumer durables and size of land holding. This study then retained the wealth index as recorded in the original survey 5 groups (lowest, second, middle, fourth, highest).

### Analytical approach

The Stata survey module ('svy') was used to account for sampling weights, stratification, and clustering. The prevalence was estimated using percentage. Forest plot analysis was conducted to determine the heterogeneity of stunting, anemia and exclusive breastfeeding across the study countries. Forest plot is necessary to synthesize data in an observational study. Stata software does not have limitations in dealing with descriptive data, and the graphical display of summary statistics such as prevalence. In addition, we computed each weighted effect size (w*es) in the forest plot. This is computed by multiplying each effect size by the study weight. The *Q* test measures heterogeneity among countries, and works like a *t* test. It is calculated as the weighted sum of squared differences between individual study effects and the pooled effect across countries, with the weights being those used in the pooling method. *Q* is distributed as a chi-square statistic with k (number of countries) minus 1 degrees of freedom. Our null hypothesis is that all countries are equal. We reject the null hypothesis at *p* < 0.05 (and hence the countries estimates are not similar). Statistical significance was determined at 5%. Stata 14 (StataCorp, College Station, TX, USA) was used.

### Ethical approval and informed consent

This study was based on an examination of population-based datasets in the public domain and freely available online, with no identifying information. MEASURE DHS/ICF International gave the authors permission to use the data. The DHS Program adheres to industry standards for preserving the privacy of respondents. ICF International assures that the survey complies with the Human Subjects Protection Act of the United States Department of Health and Human Services. Before conducting the surveys, the DHS team requested and received ethical approval from each country’s National Health Research Ethics Committee (HREC). This study did not require any additional approvals. Further information on data and ethical standard can be found here: http://goo.gl/ny8T6X.

## Results

Table 1 shows the list of study countries and the year of survey. The surveys were conducted from 2002–2020. See Table 1 below for the details.

**Table 1:** Demographic and Health Surveys conducted from 2002–2020

Country	Year of survey
Angola	2015–16
Benin	2017–18
Burkina Faso	2010
Burundi	2016–17
Cameroon	2018
Chad	2014–15
Comoros	2012
Congo	2011–12
Congo Democratic Republic	2013–14
Cote d’Ivoire	2011–12
Egypt	2014
Eritrea	2002
Eswatini	2006–07
Ethiopia	2019
Gabon	2012
Gambia	2019–20
Ghana	2014
Guinea	2018
Kenya	2014
Lesotho	2014
Liberia	2019–20
Madagascar	2008–09
Malawi	2015–16
Mali	2018
Morocco	2003–04
Mozambique	2011
Namibia	2013
Niger	2012
Nigeria	2018
Rwanda	2019–20
Sao Tome and Principe	2008–09
Senegal	2019
Sierra Leone	2019
South Africa	2016
Tanzania	2015–16
Togo	2013–14
Uganda	2016
Zambia	2018
Zimbabwe	2015

**Fig. 1:**
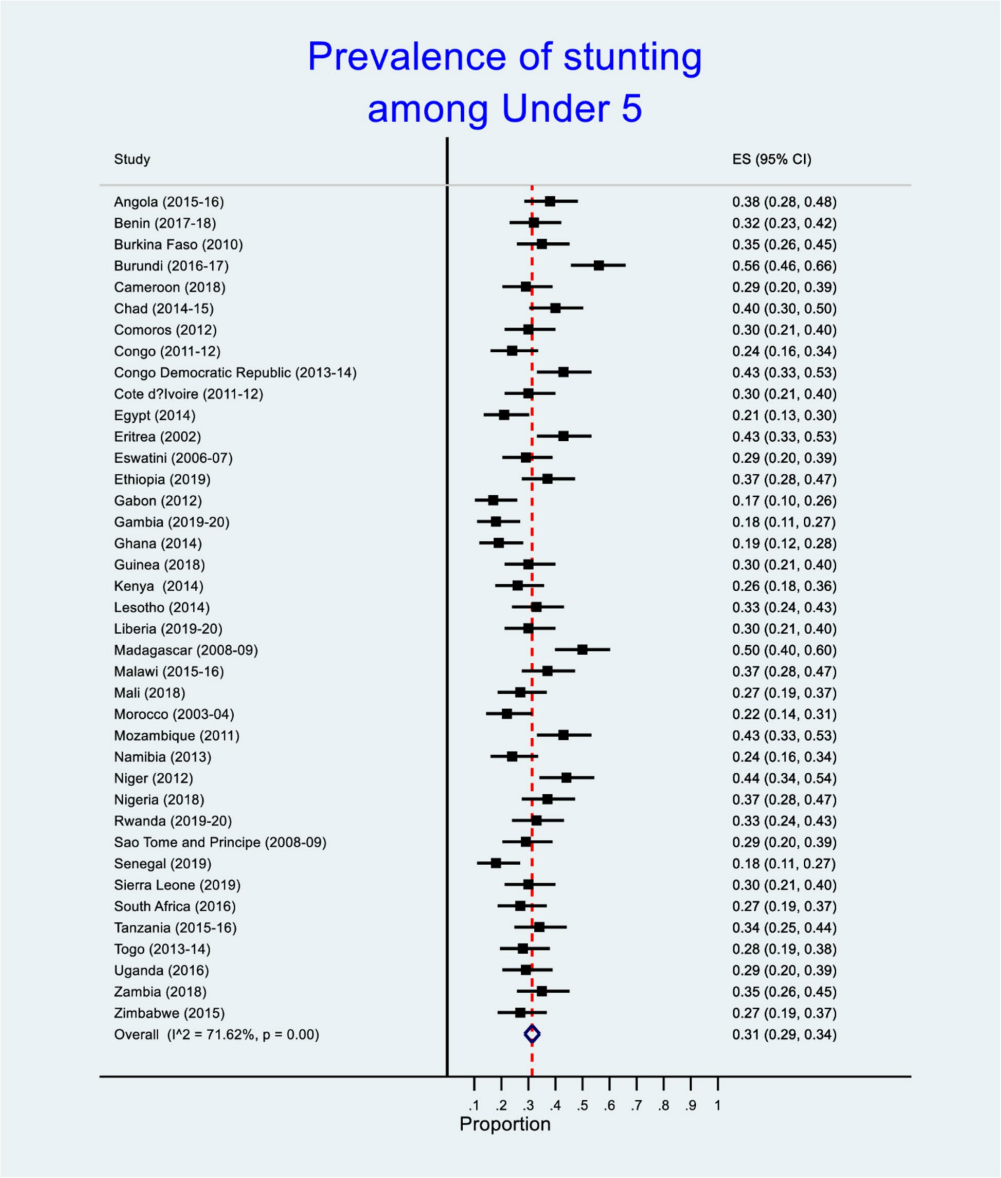
Forest plot with effect size (ES) for stunting among under 5 children in African countries

Figure 1 shows the prevalence of stunting among African children. The prevalence of stunting was highest in the following countries: Burundi (56%), Madagascar (50%), Niger (44%), Congo Democratic Republic, Eritrea and Mozambique (43% each) and Chad (40%) respectively. On the other hand, Gabon, Gambia and Ghana had 17%, 18% and 19% of under 5 stunting respectively. We found statistical significant difference in the prevalence of under 5 stunting across African countries (*p* < 0.001). See Fig. 1 for the details.

**Fig. 2:**
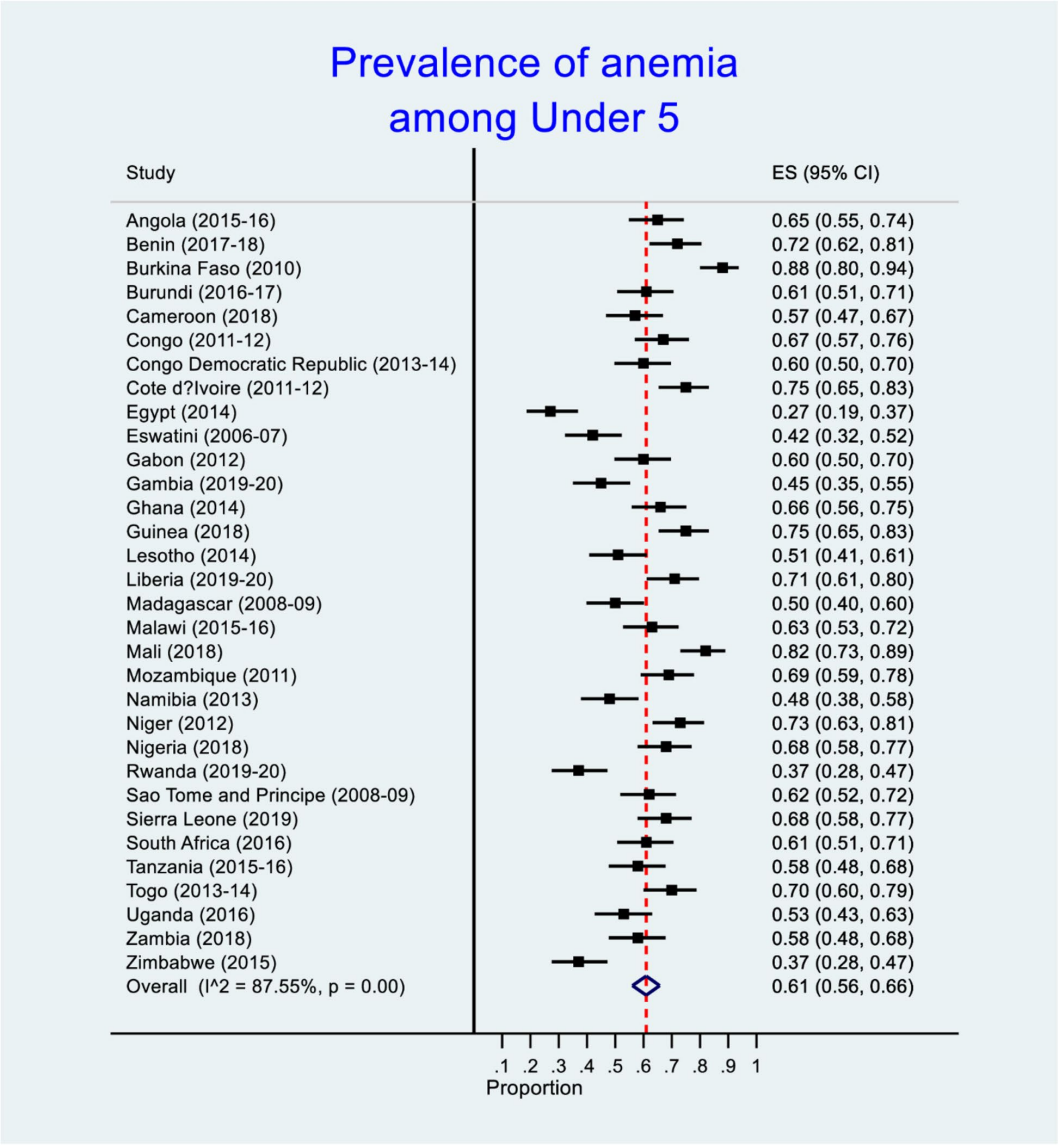
Forest plot with effect size (ES) for anemia among under 5 children in African countries

Figure 2 shows the prevalence of anemia among under 5 African children. The prevalence of anemia was highest in the following countries: Burkina Faso (88%), Mali (82%), Cote d’Ivoire and Guinea (75% each), Niger (73%), Benin (72%), Liberia (71%) and Togo (70%). There was statistical significant difference in the prevalence of under 5 anemia across African countries (*p* < 0.001). See Fig. 2 for the details.

**Fig. 3:**
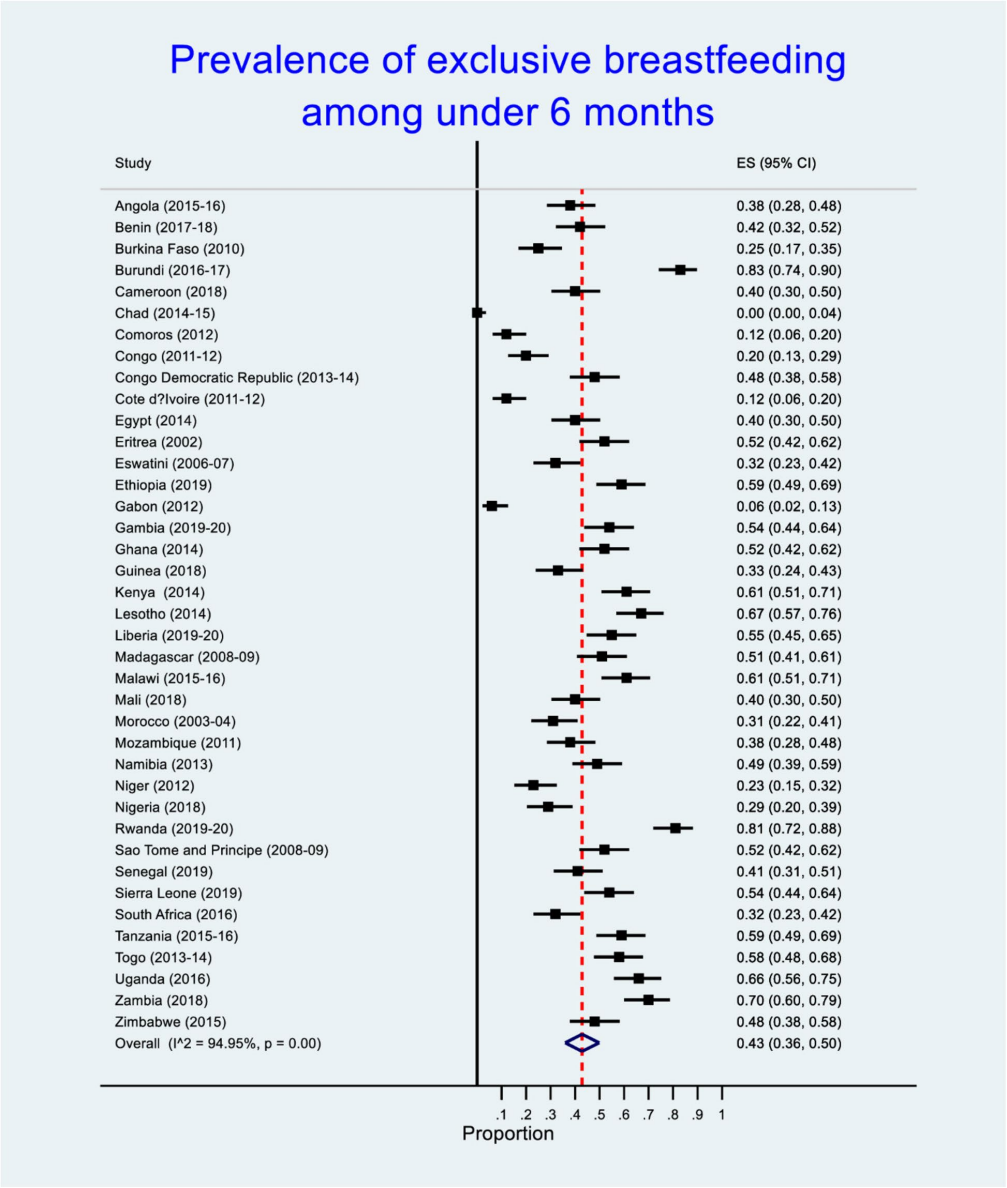
Forest plot with effect size (ES) for exclusive breastfeeding in African countries

Figure 3 shows the prevalence of exclusive breastfeeding among African children. Burundi (83%), Rwanda (81%) and Zambia (70%) had the highest exclusive breastfeeding. We found statistical significant difference in the prevalence of exclusive breastfeeding across African countries (*p* < 0.001).

Results from Table 2 shows that male children were more stunted than the female children across all countries. The children from rural residence were more stunted when compared with their urban counterparts. Furthermore, children from mothers with no formal education or primary education were more stunted than their counterparts from mothers with secondary or higher education. The children from low household wealth quintiles were more stunted than those from high household wealth group. Children with short preceding birth intervals (less than 24 months and 24–47 months) were more stunted that their counterparts who are first born or 48 + months preceding birth intervals in many African countries. See the details in Table 2 below.

**Table 2:** Distribution of stunting among under 5 children in African countries; using DHS 2002–2020

Country	Sex (%)		Residence (%)		Mother’s education (%)		Wealth quintile (%)					Birth interval (%)			
	**Male**	**Female**	**Urban**	**Rural**	**No education or primary**	**Secondary or higher**	**Lowest**	**Second**	**Middle**	**Fourth**	**Highest**	**First birth**	**Less than 24 months**	**24–47 months**	**48 + months**
Angola	41.0	34.1	31.8	45.7	42.8	24.5	47.3	45.1	38.8	26.6	20.4	35.0	46.4	37.8	27.5
Benin	35.1	29.1	27.5	35.2	33.4	22.3	41.2	38.2	32.9	28.5	18.6	31.8	35.6	32.9	24.3
Burkina Faso	36.8	32.3	21.3	37.3	35.7	10.8	41.9	37.0	37.6	33.2	18.6	35.3	40.2	35.8	27.1
Burundi	59.4	52.4	27.8	58.8	58.8	31.3	69.1	63.7	60.2	49.7	31.2	55.0	55.9	58.1	49.3
Cameroon	31.2	26.6	19.8	36.2	35.4	20.0	40.4	38.7	27.8	20.2	9.1	25.6	38.9	29.5	22.1
Chad	41.0	38.8	32.4	41.6	41.2	25.6	41.2	39.8	40.4	44.7	31.5	36.2	47.7	38.2	34.3
Comoros	32.0	28.3	24.9	32.1	33.1	21.6	38.2	32.5	25.9	27.0	21.9	29.7	38.0	27.8	23.0
Congo	25.1	23.7	20.3	30.4	28.8	19.6	34.5	27.6	26.9	17.0	9.3	22.5	32.9	25.6	16.3
Congo Democratic Republic	45.2	40.2	32.5	47.1	48.3	32.5	49.7	48.3	45.8	41.4	22.9	41.2	48.4	42.4	34.7
Cote d’Ivoire	32.7	26.9	20.5	34.9	31.2	16.9	38.1	36.1	26.3	23.8	15.6	32.6	32.1	30.6	25.0
Egypt	22.8	19.9	23.0	20.7	25.3	20.1	24.1	23.1	18.1	20.0	23.4	21.2	23.1	21.6	20.7
Eritrea	44.4	41.4	31.7	48.6	46.1	18.9	50.1	50.3	48.5	39.2	21.0	34.7	47.3	46.0	38.4
Eswatini	32.2	25.6	23.1	30.0	34.5	21.7	38.3	32.3	26.5	25.3	16.6	27.4	34.2	29.4	22.0
Ethiopia	39.8	33.6	26.2	40.4	39.2	19.5	43.3	38.6	42.2	35.2	22.6	35.5	42.7	39.7	29.6
Gabon	19.0	13.9	14.1	28.5	21.6	13.1	29.9	18.8	12.3	11.9	5.8	14.8	18.0	20.0	11.3
Gambia	18.5	16.4	16.3	19.7	17.6	16.1	22.5	19.5	15.7	13.4	15.3	14.5	19.1	18.3	15.2
Ghana	20.4	17.0	14.8	22.1	23.5	12.8	24.8	25.5	17.9	14.4	8.5	16.9	28.7	18.9	14.3
Guinea	33.8	26.8	21.7	33.8	32.5	20.9	38.0	32.8	31.4	25.3	19.3	28.7	39.9	32.4	24.7
Kenya	29.7	22.3	19.8	29.1	29.9	17.3	35.9	30.2	25.4	20.7	13.8	21.8	32.6	29.6	21.0
Lesotho	38.8	28.1	27.3	35.1	38.5	27.2	45.6	38.1	34.8	28.2	13.4	30.9	46.9	39.0	25.4
Liberia	31.8	27.9	25.0	35.1	31.7	17.0	37.9	34.7	31.3	24.3	13.6	30.6	40.8	30.9	21.7
Madagascar	52.9	47.3	43.4	50.9	51.3	46.3	47.6	54.0	52.5	51.0	43.6	47.6	59.7	50.4	44.7
Malawi	39.0	35.4	25.0	38.9	38.7	28.1	45.7	40.4	36.8	33.1	24.3	36.5	44.4	36.8	34.1
Mali	28.0	25.7	16.9	29.4	29.1	13.1	33.4	32.4	29.3	23.2	12.8	26.4	30.0	27.2	22.3
Morocco	23.9	21.0	16.2	28.8	24.9	13.5	35.0	24.8	19.7	14.7	12.5	19.3	29.3	25.7	19.6
Mozambique	44.7	40.5	35.0	45.5	44.9	27.0	51.1	48.0	46.4	37.4	24.1	46.3	45.5	43.6	36.0
Namibia	26.5	20.9	16.7	27.8	30.4	18.3	31.3	28.7	24.1	16.7	8.7	21.7	22.8	25.9	19.0
Niger	45.8	41.9	29.6	45.9	44.1	22.7	46.9	48.0	41.8	46.7	34.5	44.6	51.1	41.7	35.6
Nigeria	39.4	34.2	26.8	44.8	49.2	21.2	55.4	49.4	37.8	26.9	16.8	30.1	42.6	38.6	30.9
Rwanda	37.0	29.2	19.8	35.8	37.0	20.5	48.5	40.5	32.8	28.6	10.7	30.5	36.9	35.1	31.2
Sao Tome and Principe	29.1	29.5	29.3	29.3	31.0	24.6	38.2	34.9	32.2	20.5	17.6	27.5	38.0	32.0	25.7
Senegal	18.9	16.8	11.5	21.4	18.6	13.3	27.1	20.7	13.2	14.0	9.3	18.3	19.6	18.0	15.6
Sierra Leone	32.1	26.8	24.5	31.9	31.2	24.3	32.7	32.3	31.2	23.4	24.1	28.5	34.4	30.7	25.3
South Africa	29.8	25.0	25.7	29.2	40.4	24.8	36.3	29.4	23.9	24.5	12.5	24.0	27.1	31.0	26.6
Tanzania	36.7	32.2	24.7	37.8	35.9	23.3	39.9	39.4	38.7	29.7	19.2	33.7	35.5	36.8	28.9
Togo	28.1	26.9	16.0	33.3	29.1	17.9	33.4	37.5	32.5	19.4	10.6	24.8	35.6	28.4	21.5
Uganda	30.9	26.9	23.5	30.2	31.2	19.9	32.3	33.2	33.0	27.2	16.7	28.0	32.5	28.8	21.5
Zambia	38.3	31.0	32.1	35.9	37.6	29.9	40.1	36.6	32.9	35.3	23.9	35.0	42.1	35.1	30.5
Zimbabwe	29.6	24.0	22.1	28.5	31.8	23.4	33.0	28.8	25.4	26.3	16.6	24.3	27.3	31.9	21.3

Table 3 shows that male children were more anemic than the female children across several African countries. The children from rural residence were more anemic when compared with their urban counterparts. Furthermore, children from mothers with no formal education or primary education were more anemic than their counterparts from mothers with secondary or higher education. The children from low household wealth quintiles were more anemic than those from high household wealth group in African countries. See the details in Table 3 below.

**Table 3:** Distribution of anemia among under 5 children in African countries; using DHS 2002–2020

Country	Sex (%)		Residence (%)		Mother’s education (%)		Wealth quintile (%)				
	**Male**	**Female**	**Urban**	**Rural**	**No education or primary**	**Secondary or higher**	**Lowest**	**Second**	**Middle**	**Fourth**	**Highest**
Angola	66.2	63.2	64.5	65.2	66.7	61.9	65.5	63.1	66.8	64.8	62.3
Benin	72.0	71.0	66.1	74.8	73.2	66.0	79.3	74.5	76.5	68.0	57.9
Burkina Faso	88.6	87.0	77.6	89.9	88.7	72.2	89.1	91.0	91.5	87.9	76.0
Burundi	62.7	59.3	47.9	62.3	62.6	45.7	70.7	67.8	60.5	55.5	46.9
Cameroon	59.2	55.5	50.2	63.0	62.7	51.5	65.5	62.4	57.9	51.2	44.0
Congo	66.1	67.3	67.9	65.1	69.2	65.6	65.7	73.7	69.5	66.2	54.4
Congo Democratic Republic	61.2	58.4	58.8	60.3	60.1	60.0	65.7	60.2	61.3	55.8	53.4
Cote d’Ivoire	74.9	74.7	67.2	79.3	76.8	66.1	80.2	81.1	75.9	68.4	62.2
Egypt	27.2	27.3	23.1	29.2	28.0	27.1	34.0	32.9	23.8	25.3	21.3
Eswatini	43.5	40.1	50.0	40.4	43.7	44.8	43.4	38.7	44.0	43.2	39.4
Gabon	61.0	59.4	59.4	64.6	65.0	59.1	64.7	63.2	59.0	58.5	53.4
Gambia	47.0	42.3	37.1	59.5	49.9	37.7	63.6	45.3	40.2	41.0	29.9
Ghana	65.5	66.0	58.3	72.0	75.3	58.7	79.4	74.9	63.8	58.3	47.2
Guinea	75.4	73.8	71.0	76.1	75.6	72.7	73.0	81.2	73.1	74.3	69.8
Lesotho	52.8	48.9	48.3	51.6	51.4	56.8	53.9	54.8	50.8	47.1	45.0
Liberia	70.8	70.8	70.1	71.5	71.1	70.9	70.3	71.8	76.5	69.8	63.3
Madagascar	51.7	48.9	47.6	50.6	50.7	47.5	57.1	51.9	49.3	47.2	40.1
Malawi	62.9	62.3	56.1	63.5	64.3	58.2	68.2	65.8	59.9	61.1	54.2
Mali	82.4	81.3	75.1	83.6	84.0	72.4	85.7	85.7	84.9	79.5	70.9
Mozambique	69.0	68.3	59.7	72.0	71.2	54.2	77.8	76.3	68.3	62.7	51.5
Namibia	49.6	45.4	46.6	48.0	50.6	48.8	49.3	50.6	48.4	44.6	40.8
Niger	74.5	72.3	69.8	73.9	73.9	68.0	76.3	70.4	75.9	74.3	69.1
Nigeria	69.5	66.2	62.0	72.5	73.8	61.1	80.1	75.0	66.5	65.3	53.3
Rwanda	38.0	35.1	34.0	37.1	37.2	35.4	41.8	37.0	37.1	35.2	30.4
Sao Tome and Principe	64.8	59.6	66.2	58.5	64.5	62.1	65.6	65.7	62.9	57.9	57.8
Sierra Leone	71.1	64.5	57.0	73.3	71.8	61.5	73.0	74.8	70.3	62.7	50.9
South Africa	63.9	58.7	62.2	60.4	60.8	62.5	63.6	62.2	59.6	60.3	57.6
Tanzania	59.5	56.0	53.5	59.2	59.3	55.2	63.7	60.1	58.4	52.6	50.1
Togo	70.9	63.3	63.5	73.4	73.4	61.6	71.8	76.0	73.6	67.4	59.4
Uganda	53.7	51.8	47.7	54.0	55.5	49.3	65.6	54.4	48.7	48.5	44.8
Zambia	59.7	56.6	58.1	58.2	58.8	59.5	61.0	57.4	57.8	56.8	56.8
Zimbabwe	37.6	35.9	37.5	36.5	38.9	37.9	39.9	32.4	36.4	35.0	0.4

## Discussion

We explore the prevalence of stunting, anemia and exclusive breastfeeding amongst children in African countries. Our findings show differences in stunting, anemia and exclusively breastfeeding across African countries. The prevalence of stunting varied from 17% in Gabon to 56% in Burundi. Most African countries have high prevalence of stunting, signifying an indication of public health challenge in Africa. Based on the WHO cut-off value for public health importance, the estimate of many countries falls in the high prevalence category ($\ge$ 30%) [34]. In addition, we observed that stunting has a higher prevalence among rural children in African countries, when compared with urban children. This is consistent with reports from previous studies in developing countries [35–38]. A possible explanation to the findings could be due to low wealth-related and mother’s education status, inadequate water supply, incidence of infectious disease and poor knowledge of nutrition, which is more prevalent in rural residence, when compared with urban counterparts [39]. In addition, mothers in the rural areas are more burdened with inadequate knowledge of infant and young child feeding practices and inadequate access to health care services [40, 41].

We found higher prevalence of stunting among male children and those with less than 24 months preceding birth interval. It seems stunting may be carried on from in utero differential growth trajectories by gender [42]. The estimates of higher prevalence of stunting among male children is in agreement with results from previous studies [36, 37, 43–45]. Although the reason for the sex differences in stunting is not well-known, however, there is indication that it may be due to differences in feeding and care practices between both gender [44]. Furthermore, the high prevalence of stunting among children with less than 24 months preceding birth interval, has been reported from previous studies [43, 46]. Short birth interval may be linked to improper feeding practices. The high prevalence of stunting among children in less than 24 months preceding birth interval is consistent with the findings from previous studies [46, 47]. This is likely especially among disadvantaged children. Another explanation could be mother’s inability to care for multiple children at the same time with limited household resources including food.

Our study found that mother’s education and higher household wealth status and urban residence played a significant role in lower prevalence of stunting among under 5 children. This is consistent with the findings from a previous study [48]. Access to clean potable water in higher wealth households and urban residence may have resulted to lower prevalence of stunting among the well-off. A child from a lower-educated mother suffers more from stunting than a child of a higher-educated mother. These findings are in agreement with results from previous studies [35, 36, 38, 43, 49, 50]. Educated mothers could have a better understanding of child nutrition, proper child care, hygiene, uptake of health services and they are more likely to seek expert opinion on child well-being and development. Children who came from the richest households were less likely to suffer from stunting than those from the poorest household. This corroborates with previous studies [38, 46, 51].Children from low-socioeconomic households have more likelihood to be exposed to poor nutrition, which could lead to stunting when compared with those from high socioeconomic households. The plausible explanation is the negative effect of low socioeconomic status in access to food or good nutrition, hence their uptake of adequate nutrient is low [52]. On the other hand, individuals with higher socioeconomic status can afford proper nutrition, which supports healthy living and improved child care. Notably, this leads to a reduction in malnutrition. The high prevalence of stunting reported in this study suggests more investments in social protection programmes that particularly target households with young children, which is necessary to address the high burden of stunting in African countries.

Furthermore, this study show that the prevalence of anemia among under 5 children is worrisome, ranging from 27% in Egypt to 88% in Burkina-Faso indicating that anemia is still a major public health concern in African countries. It shows that efforts in the implementations of programmes and strategies in managing infectious diseases, including deworming, malaria and iron supplementation in the region have not paid off adequately. According to the WHO classification of anemia as a public health issue, it can be severe, moderate and mild, when the prevalence is more than 40%, 20%, 5% respectively [53]. Therefore, the prevalence of anemia among under 5 children as found in this study, would be classified as severe according to the WHO cut-off point. Many previous studies have reported similar findings [19, 54, 55]. This could be due to the fact that many children were not exclusively breastfeed and the practice of inadequate feeding. Several reports have shown that poor breastfeeding practices and poor dietary diversity are linked to childhood anemia [56, 57]. Thus, when complementary feeding, such as cow milk, is timely introduced before 6 months of age, they could not substitute for iron rich foods, and thereby may lead to iron deficiency anemia. Also, malaria incidence, hookworm infestation, schistosoma and visceral leishmaniasis infection due to lack of proper sanitation and better environmental conditions could contribute to high prevalence of anemia [54].

We observed a high prevalence of anemia among rural dwellers, when compared with their urban counterparts and among male than the female children. The high prevalence of anemia among rural dwellers has been reported in similar settings [38, 58]. This could be attributed to malnutrition due to limited consumption of nutritious foods because of poverty and low socioeconomic status, poor sanitation facilities and lack of good drinking water [58], leading to increased rates of diseases and infections and subsequently increased risk of anemia. Our results mirrored findings from previous and similar studies, where male children have higher prevalence of anemia than their female counterparts [51, 54, 55, 59]. This may be due to the rapid growth and development of male children in the first few years of life, that increases their micronutrients requirement including iron than the female children [54].

This study also found socioeconomic status (wealth and education) of mother’s influence on childhood anemia. Children from low household wealth and those whose mothers had no formal education or primary education had higher prevalence of anemia than children from higher household wealth and those whose mothers had secondary and higher educational level. Previous studies reported similar findings [51, 54–56, 59, 60]. The possible explanation is that poor household wealth status might restrict families to access available health services, good sanitation facilities and the ability to purchase nutritious and healthy foods. Moreover, children from low socioeconomic households are more likely to experience food insecurity. Additionally, lower educated mothers are less likely to be knowledgeable about proper care for their own health and that of their children. Mother’s education has great influence in feeding practices of children and proper health care. Mothers who are educated are very conscious of their child’s health and they follow guidelines on proper feeding practices, which tends to improve their child's nutritional status.

The prevalence of exclusive breastfeeding in this study varied largely across African countries, ranging from 6% in Gabon to 83% in Burundi. This is line with other previous studies in similar settings and other regions [61–63]. A possible explanation could be dearth of knowledge about optimal breastfeeding practices. It is of utmost importance to increase communal-based behaviour change communication by means of several channels such as media and radio to educate mothers on the importance of optimal newborn and young feeding practices since suboptimal and child undernutrition remains a major issue in African countries. It is critical to focus urgently on improving children health and nutrition, particularly in rural areas and low socioeconomic status, in order to attain the SDGs aiming at zero hunger and the eradication of all kinds of malnutrition. To minimize childhood stunting and anemia in African countries, policies should focus on nutrition-specific interventions, including exclusive breastfeeding, optimal feeding practices, nutritional supplementation and child awareness-related activities, which should primarily target rural and underserved populations. Improving the nutritional status of under 5 children requires concerted efforts from both government and non-governmental organizations.

### Strengths and limitation

This study has presented estimates of stunting, anemia and exclusive breastfeeding in African countries. Nationally representative large datasets were analyzed for plausible comparisons. The ability to pool many countries is a major advantage. This study can be used as a scorecard for various countries and to indicate the performance of healthcare system in various countries. This can instigate concerted efforts and new policies and programmes, as well as a call to strengthen existing programmes related to proper child feeding practices and action against hunger and poverty in general. This study would bring to limelight a call for other low and middle income countries (LMICs) to examine the uptake of exclusive breastfeeding and burden of stunting and anemia amongst others. However, we used a cross-sectional study to collect data from various countries at various points in time. Some has quite long different period (example: Eritrea was derived in 2002, whereas some countries were derived in 2017 to 2020). It might have potential factors influencing the socioeconomic condition of each country that could be linked to the variables of the study. These factors including political situation, development of health care facilities and the government’s health-policy which could lead to different capture of socioeconomic condition in each country in different period of time. As a result, the distribution of stunting and anemia may have shifted over time. This may results in sampling bias. Furthermore, the DHS does not collect data on household income or expenditure, which are traditional indicators of wealth. The assets-based wealth index used here is only a proxy for household economic status, and it does not always produce results that are comparable to those obtained from direct measurements of income and expenditure where such data are available or can be collected reliably. In addition, we do not know the proportion of stunted and anemic children, whether it is due to genetics or purely malnutrition, as other factors could have also contributed.

## Conclusion

This study show high prevalence of stunting and anemia among under 5 children in Africa, particularly in rural areas and among the disadvantaged. Other factors such as being male, poor household wealth, short birth interval, low mother’s education are linked with high prevalence of stunting and anemia among under 5 children. In addition, there were sub-optimal exclusive breastfeeding practice among mothers in the studied countries. To reduce stunting and anemia among under 5 children, national public health intervention programmers and stakeholders working on improving childhood nutrition should focus on these factors. Mothers or caregivers should be educated about the benefits of exclusive breastfeeding, proper feeding practices, women’s empowerment and adequate birth spacing. Programme planners and policymakers should evaluate and increase collaboration and coordination of nutritional programmes and family health programmes targeted at alleviating nutritional inadequacies.

## Data Availability

Data for this study were sourced from Demographic and Health surveys (DHS) and available here: http://dhsprogram.com/data/available-datasets.cfm.
